# A Meta-Analysis of the Association between the hOGG1 Ser326Cys Polymorphism and the Risk of Esophageal Squamous Cell Carcinoma

**DOI:** 10.1371/journal.pone.0065742

**Published:** 2013-06-06

**Authors:** Junjie Zhang, Jingshi Zhou, Ping Zhang, Weiping Wang, Shiheng Tao, Minghua Wang

**Affiliations:** 1 College of Life Science, Northwest A & F University, Yangling, Shaanxi, China; 2 Department of Biochemical and Molecular Biology, Medical College, Soochow University, Suzhou, Jiangsu, China; 3 Department of Hepatopancreaticobiliary Surgery, Xijing Hospital, the Fourth Military Medical University, Xi'an, Shaanxi, China; 4 Bioinformatics Center, Northwest A & F University, Yangling, Shaanxi, China; New Jersey Institute of Technology, United States of America

## Abstract

**Background:**

Genetic polymorphism of human 8-oxoguanine glycosylase 1 (hOGG1) Ser326Cys (rs1052133) has been implicated in the risk of Esophageal Squamous Cell Carcinoma (ESCC). However, the published findings are inconsistent. We therefore performed a meta-analysis to derive a more precise estimation of the association between the hOGG1 Ser326Cys polymorphism and ESCC risk.

**Methodology/Principal Findings:**

A comprehensive search was conducted to identify eligible studies of hOGG1 Ser326Cys polymorphism and the risk of the ESCC. Three English and two Chinese databases were used, and ten published case-control studies, including 1987 cases and 2926 controls were identified. Odds ratios (ORs) and 95% confidence intervals (CIs) were used to assess the strength of the association in the dominant and recessive model. Pearson correlation coefficient (PCC) and standard error (SE) were used to assess the number of Cys allele and ESCC risk in the additive model. Overall, significant associations between the hOGG1 Ser326Cys polymorphism and ESCC risk were found in the recessive model: OR = 1.37 (95% CI: 1.06–1.76, p = 0.02). We also observed significant associations in the Caucasian, Chinese language, population based control and tissue subgroups. In the additive model, positive correlation was found between the number of Cys allele and the risk of ESCC in overall studies (PCC = 0.109, SE = 0.046, p = 0.02), Caucasian subgroup and population subgroup. Funnel plot and Egger's test indicate there was no publication bias in this meta-analysis.

**Conclusion:**

Under the published data, the hOGG1 Ser326Cys polymorphism is associated with ESCC risk in the recessive and additive model. Compared with the Ser/Ser and Ser/Cys genotype, Cys/Cys genotype might contribute to increased risk of ESCC. And the risk of ESCC is positively correlated with the number of Cys allele. A better case-control matched study should be designed in order to provide a more precise estimation.

## Introduction

Esophageal cancer (EC) is the sixth most common cancer worldwide with 5-year survival rate less than 10% and occurs at a relatively high frequency in certain areas of China [1], [2]. Esophageal Squamous Cell Carcinoma (ESCC) is the major type of EC in China, and the mechanism of ESCC still remains unclear. Many factors may increase the risk of ESCC, including environmental and genetic factors. Several studies have found out that DNA repair efficiency in cancer patients is lower than that of normal people, and the variants of the genes involved in DNA repair can lead to increasing risk of cancer [3]. The human 8-oxoguanine glycosylase 1 (hOGG1) gene, located on chromosome 3, encodes 8-hydroxygumine DNA glycosylase 1 (OGG1) that can repair damaged DNA by excising 8-dihydro-8-oxoguanine (8-OH-G) [4]. Genetic variations in hOGG1 gene may alter glycosylase activity, increasing the cancer risk [5]. There are several polymorphisms in the hOGG1 gene [6]; and Ser326Cys polymorphism has attracted widespread attention. With a Ser to Cys amino acid substitution at codon 326, Ser326Cys can affect the function of hOGG1. This variation maybe associated with risk of cancer. A study indicated that compared to the 326Cys variant enzyme, the 326Ser enzyme of hOGG1 has a higher activity [7]. Many studies have focused on the association of Ser326Cys polymorphism in hOGG1 and ESCC risk, including several case-control studies [8]–[12]. However, the results of these studies remained inconclusive and inconsistent. In this meta-analysis, by searching individual dataset from all eligible case-control studies published to date, we aimed to estimate the role of hOGG1 Ser326Cys polymorphism in the risk of ESCC as well as to quantify the between-study heterogeneity and potential bias.

## Materials and Methods

### Identification and Eligibility of Relevant Studies

To identify all published studies that examined the association of hOOG1 Ser326Cys polymorphism with ESCC risks, we conducted a computerized literature search of following databases: PubMed, Web of Science, Medline, China Knowledge Resource Integrated Database (CNKI) and Wanfang database. The key words were as follows: (“esophageal cancer” OR “oesophageal cancer” OR “ESCC”), (“OGG1” OR “hOGG1” OR “8-Oxoguanine DNA glycosylase 1”), and (“polymorphism” OR “variation” OR “mutation” OR “SNP”). In the CNKI and Wanfang, corresponding Chinese characters of the keywords were used for searching. References of retrieved articles were also screened. When a study reported results on different subgroups, we treated each subgroup as a single comparison in the meta-analysis. Studies included in this meta-analysis should meet the following criteria: firstly, evaluate the association between hOGG1 Ser326Cys polymorphism and ESCC risk; secondly, use a case-control design; thirdly, contain available genotype frequency or genotype frequency can be calculated. Additionally, other relevant studies were identified by hand-searching the references of the eligible articles.

### Data Extraction

Two investigators independently extracted data and reached a consensus on all the items. For each study, the following data were included: the last name of first author, the year of publication, the DNA source of patients (from blood or tumor tissue), ethnicity, the source of controls (population- or hospital-based), genotyping method, numbers of patients and controls, genotypes distribution in each group and published language. We also tested Hardy-Weinberg equilibrium (HWE) of genotypes distribution in control groups.

### Statistical Analysis

We examined hOGG Ser326Cys genotypes under the dominant (Cys/Cys+Cys/Ser vs. Ser/Ser), recessive (Cys/Cys vs. Cys/Ser+Ser/Ser), and additive (Cys/Cys vs. Cys/Ser vs. Ser/Ser) models.

The strength of the association between the hOGG1 Ser326Cys polymorphism and ESCC susceptibility was measured by odds ratios (ORs) with 95% confidence intervals (CIs). In the dominant and recessive model, the statistical significance of the summary OR was determined with the Z test. Heterogeneity assumption was checked by the Q test [13]. If the p-value was greater than 0.1, a random effect model was used to pool the results. Otherwise, a fixed effects model was then used [14], [15]. In the additive model, in order to identify the association between the ESCC risk and the number of copies of the 326Cys allele (0, 1 or 2), Cochran-Armitage trend test was used to calculate p-value for each study [16], [17]. Greenland and Longnecker's method was used to give the result of overall studies [18].

Stratified analyses were performed by published language (English or Chinese), ethnicity (Asian or Caucasian), source of controls (population- or hospital-based) and the DNA source of patients (blood or tumor tissue).

Meta-regression models were also employed to evaluate the different variance among the individual ORs when heterogeneity was detected. Potential sources of heterogeneity were: published language, ethnicity of the population, source of control and the source of patient DNA.

In order to analyze the deviation of the single study that may denote bias to the overall results, all studies were subjected to a sensitivity analysis. If a study affected the overall result significantly, comprehensive analysis would be taken to find out the cause of heterogeneity. Once found significant heterogeneity, we would like to find out the reason that causes the heterogeneity in the following analysis. Study that deviated from HWE was also removed, for investigating the affection for the overall results. Furthermore, in order to track evidence over time, we also performed a cumulative meta-analysis, in which studies were chronologically ordered by publication year, and pooled ORs were calculated at the end of each year. Funnel plots and Egger’ s linear regression test were used to provide diagnosis of the potential publication bias [19]. Results were regarded as statistically significant if P<0.05. All statistical tests were performed with Review Manage, version 5.2 and R, version 2.14.1 using two-sided p-values.

## Results

### Characteristics of Studies

**Figure 1:**
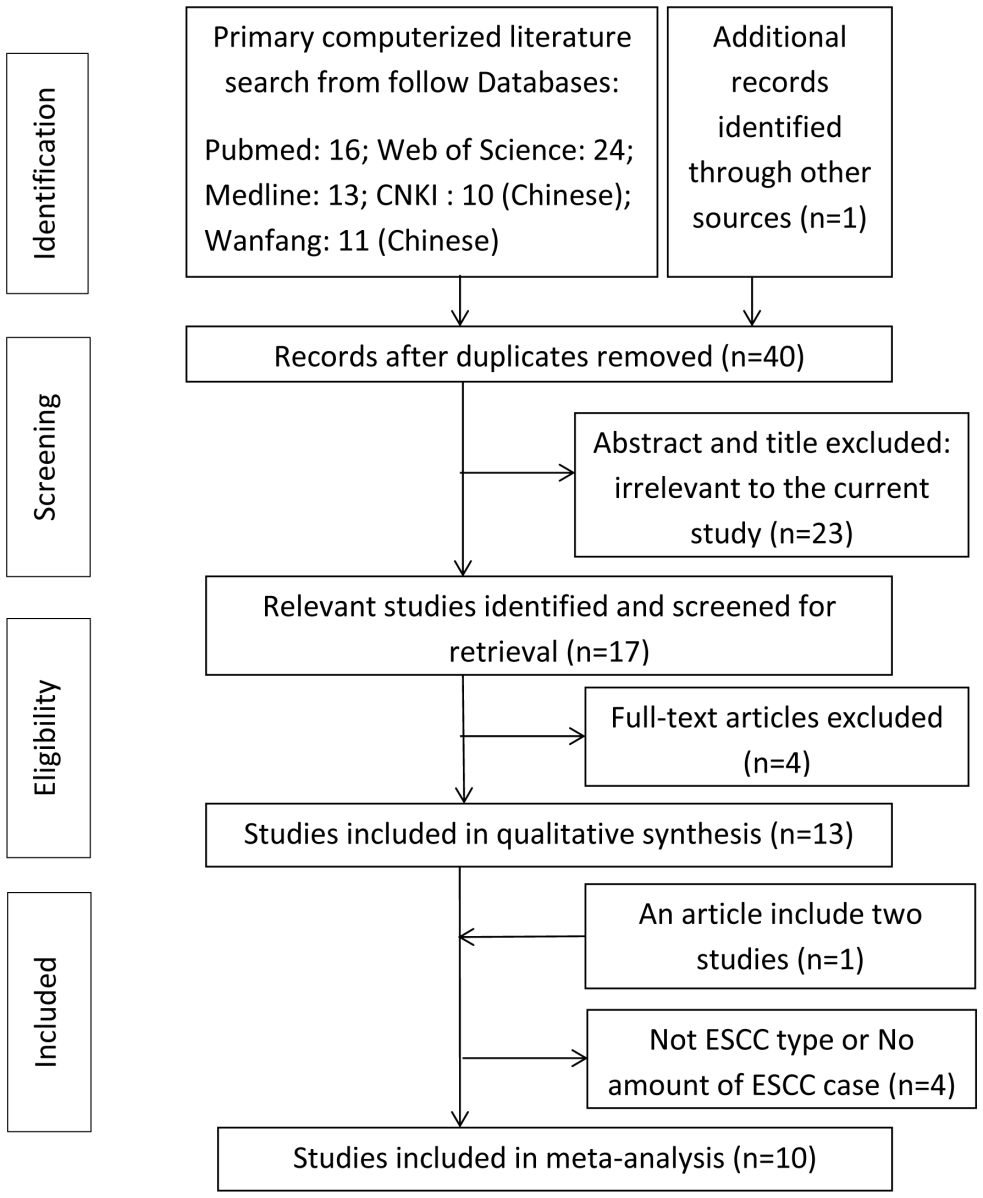
Flow diagram of the study selecting process.

There were 40 articles relevant to the search words after removing duplicates records. By reading the titles and abstracts, 23 articles were excluded. Besides, we reviewed the full texts and removed 4 articles because they are not related to our research or not case-control designs. Moreover, we found an article consists of two study groups, so we treated it as two studies in our analysis. Then we checked the type of esophageal cancer and the information about the number of ESCC patients. In this stage, we found that two studies were not related to ESCC, but esophageal adenocarcinoma (EAC) [20], [21]. Another two studies contain both types of EC: EAC and ESCC, but we can’t get the numbers of ESCC patients even after contacting authors [22], [23]. Finally, according to our inclusion criteria, a total of 10 eligible studies, including 6 English articles [8]–[12] and 4 Chinese studies [24]–[27], involving 1987 cases and 2926 controls were enrolled in the pooled analyses (Table 1). Among 4 Chinese articles, one of them only has Chinese title and abstract (we have translated the title into English) [24], others have both the title and abstract in English. The flow chart of literature search and study selection was illuminated in Figure 1. These studies were conducted in different populations of various ethnicities: 6 studies of Chinese, 1 study of European, 2 studies of Indian and 1 study of Kazakh. Control sources were from 6 population-based and 4 hospital-based. DNA of the patients were extracted from different source, 7 studies from blood and 3 studies from tissue. In addition, genotype distribution of the control group in one study was not consistent with HWE.

**Table 1:** Characteristics of studies included in this meta-analysis.

Fist author	Year	Published language	Ethnicity	Source of Controls	DNA Source of patients	Genotyping method	p for HWE	Genotypes of Cases			Genotype of Controls		
								S/S	S/C	C/C	S/S	S/C	C/C
Hall	2006	English	European^b^	Hospital	blood	Taqman	0.061	107	56	10	622	320	27
Hao	2004	English	Chinese	population	blood	PCR-RFLP	0.250	153	180	77	184	216	79
Hu	2010	Chinese	Chinese	population	tissue	PCR-RFLP	0.096	88	93	54	80	120	28
Li	2011	English	Chinese	Hospital	blood	PCR-SSCP	0.154	86	126	13	97	123	26
Liu	2005	Chinese	Chinese	Hospital	blood	PCR-CTPP	0.682	16	57	33	21	50	35
Upadhyay(K)^a^	2010	English	Indian	population	blood	PCR-CTPP	0.128	59	66	10	94	89	12
Upadhyay(U)^a^	2010	English	Indian	population	blood	PCR-CTPP	0.020	84	97	19	96	100	11
Wang	2009	Chinese	Kazakh	Hospital	blood	PCR-LDR	0.338	30	61	28	26	50	16
Xing	2001	English	Chinese	population	tissue	PCR-SSCP	0.154	78	76	42	68	106	27
Zhu	2009	Chinese	Chinese	population	tissue	PCR-SSCP	0.981	67	85	36	80	95	28

a Two groups in this article, K means Kashmiri, U means Uttar Pradesh;

b Romania, Poland, Russia, Slovakia and Czech.

**Abbreviations:** HWE: Hardy–Weinberg equilibrium; S/S: Ser/Ser; S/C: Ser/Cys; C/C: Cys/Cys.

### Quantitative Synthesis

**Figure 2:**
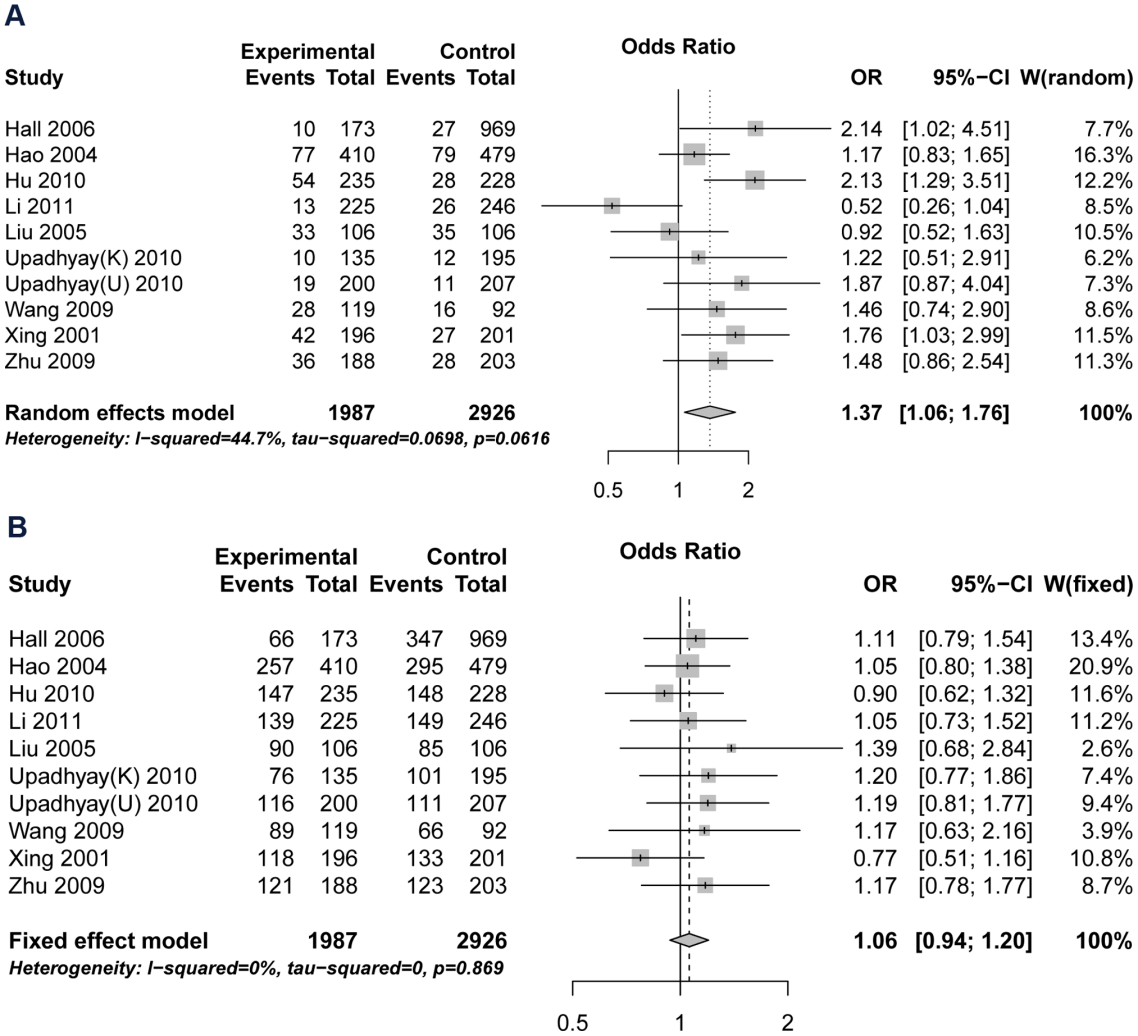
Forest plots of odds ratios (ORs) with 95% confidence limits (CIs). The center of each square represents the OR. The area of the squares reflects the weight, and the horizontal line indicates the 95% CI. (A). recessive model; (B). dominant model.

We carried out a meta-analysis of the hOGG1 Ser326Cys polymorphism overall, and in subgroups according to ethnic groups, published languages, DNA source of patients and the source of controls. Results of the recessive and dominant model are shown in Table 2 and Table S1, respectively. Forest plots are shown in Figure 2. There was a significant association between the hOGG1 Ser326Cys and ESCC risk in the overall analysis in the recessive model (OR = 1.37, 95% CI 1.06–1.76, P = 0.02). However, the result was not significant under a dominant model (OR = 1.06, 95% CI: 0.94–1.20; P = 0.36). There was significant heterogeneity between studies under the recessive model (P = 0.06; I^2^ = 45%) but not the dominant model (P = 0.87; I^2^ = 0%). In subgroup analysis, the hOGG1 Cys/Cys polymorphism was significantly associated with the risk of ESCC in the recessive model performed by ethnicity, published language, DNA source of patients and the source of control groups. We found that Cys/Cys variants in subgroups of Chinese language, Caucasian, controls of population and tissue increased ESCC risks, with the ORs of 1.49 (95% CI 1.12–1.96, p = 0.006), 1.64 (95% CI 1.12–2.40, p = 0.01), 1.50 (1.22–1.85, p = 0.0001), 1.79 (1.33–2.42, p = 0.001), respectively. However, no significant result was observed in the dominant model in any subgroup. In the additive model, pairwise comparison of genotypes and p-value of the Cochran-Armitage test for each study are shown in Table S2. Overall and subgroup results are shown in Table S3. Positive correlation was found between the number of Cys allele and the risk of ESCC (PCC = 0.109, SE = 0.046, p = 0.02). In subgroup analysis, the risk of ESCC is positively correlated with the number of Cys allele in Caucasian subgroup (PCC = 0.19, SE = 0.084, p = 0.02) and population subgroup (PCC = 0.12, SE = 0.055, p = 0.03). No heterogeneity was found between studies (P = 0.94; I^2^ = 0%).

**Table 2:** Quantitative analyses and the test of heterogeneity of the hOGG1 Ser326Cys polymorphism on the ESCC risk in a recessive model.

	Q-test						
	chi-squared	df	p-Value	I^2^(%)	sample size^a^	OR(95%CI)	p-Value
Overall	16.26	9	0.06	45	4913	1.37(1.06,1.76)	0.02^b^
Ethnic group							
Asian	13.75	5	0.02	63.6	2823	1.25(0.88,1.77)	0.21^b^
Caucasian	1.16	3	0.76	0	2090	1.64(1.12,2.40)	0.01
Published language							
English language	10.97	5	0.05	54.4	3636	1.31(0.90,1.90)	0.16^b^
Chinese language	4.7	3	0.2	36.1	1277	1.49(1.12,1.96)	0.006
Source of controls							
Population	4.75	5	0.45	0	2877	1.50(1.22,1.85)	0.0001
Hospital	8.64	3	0.03	65.3	2036	1.09(0.62,1.93)	0.77^b^
DNA source							
blood	10.37	6	0.11	42.2	3662	1.15(0.93,1.44)	0.2
tissue	0.95	2	0.62	0	1251	1.79(1.33,2.42)	0.0001

a Sample size equals the total number of controls and cases;

b Caculated by random model, otherwise by fixed model.

**Abbreviations:** OR: odds ratio; CI: confidence intervals.

Meta-regression analysis under the recessive model was performed in order to find out potential sources of heterogeneity. Empty regression was firstly run to estimate the baseline value for tau^2^ (0.070). In the meta-regression analysis, the model including DNA source of patients reduced the tau^2^ value to 0.014 (beta coefficient = 1.993, 95% CI: 0.007–0.832; P = 0.046), suggesting DNA source of patients was a significant source of heterogeneity in the recessive model.

### Sensitivity Analyses and Adjusted Results of Meta-analysis

**Figure 3:**
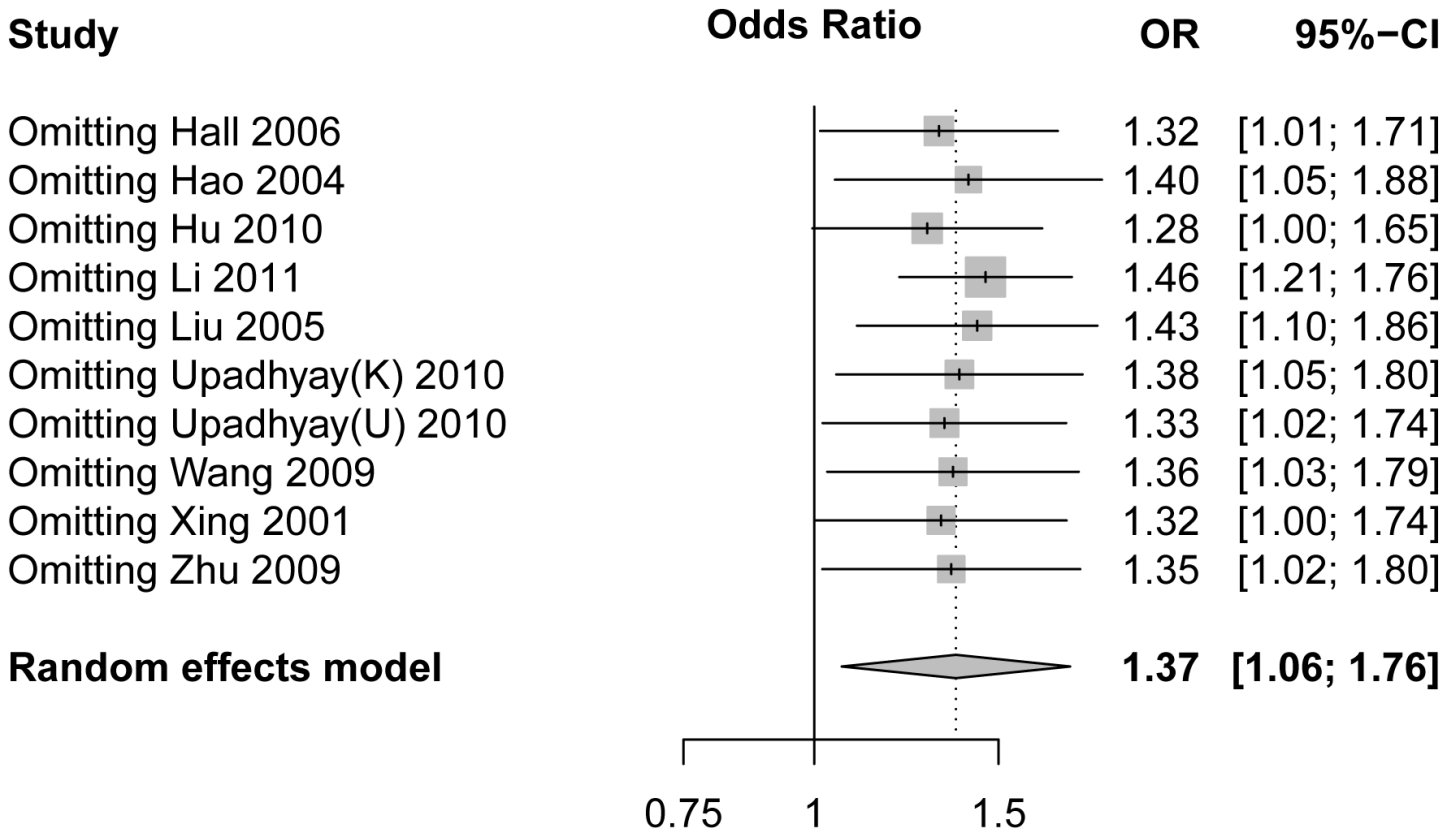
Results of odds ratios (ORs) with 95% confidence limits (CIs) in sensitivity analysis. Results were computed by omitting each study. Random-effects estimates were used. Each OR means the result when remove the corresponding study.

**Figure 4:**
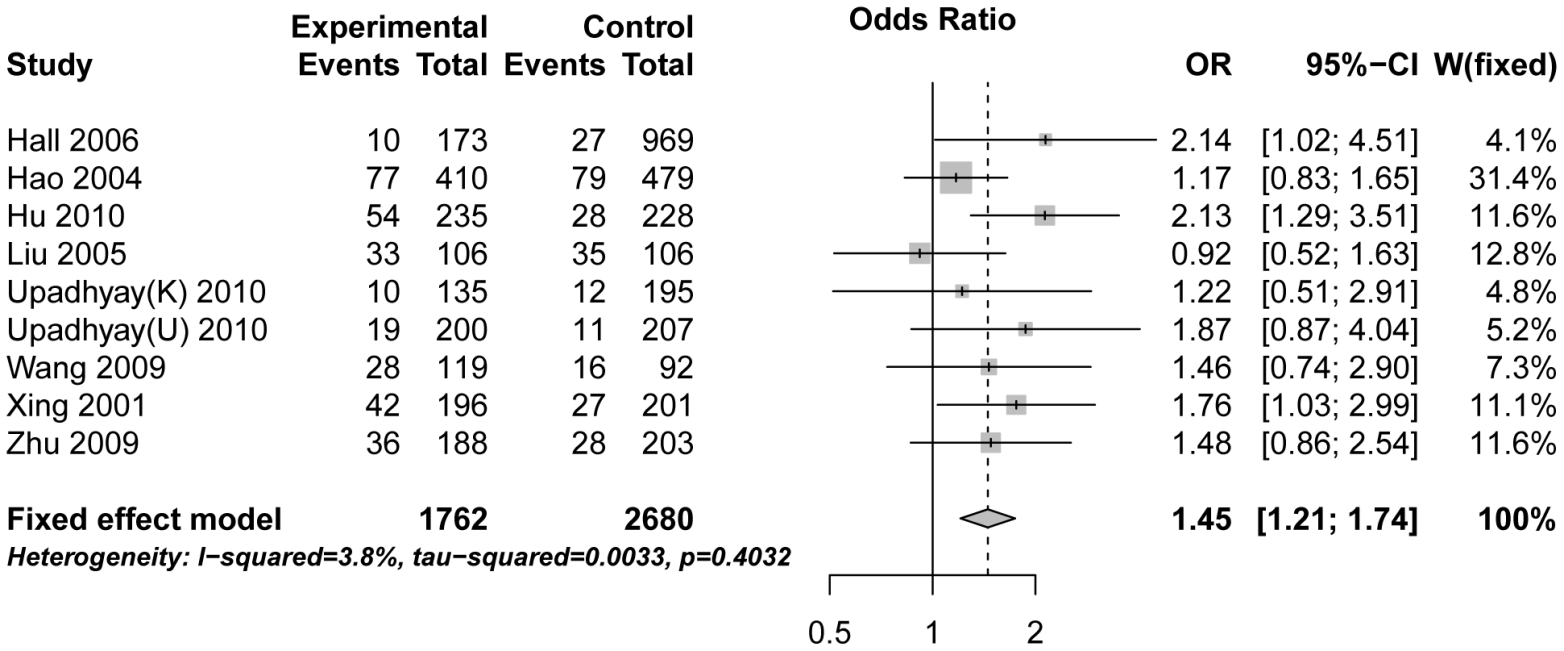
Forest plots result without the study of Li et al. The center of each square represents the OR, the area of the square is the weight used in the meta-analysis, and the horizontal line indicates the 95% CI.

Since significant heterogeneity across studies was observed for the recessive model, we conducted a sensitivity analysis to assess the influence of each individual study on the pooled OR and the heterogeneity by sequentially removing the individual study. Results are summarized in Table 3. The forest plot of sensitivity analysis in a random model is shown in Figure 3. We can see that removing the HWE-deviation study (Upadhyay 2010 “UP” population) did not affect the result significantly. We also found that the study conducted by Li et al. (2011) influents the overall pooled estimates and the heterogeneity most. We compared this study to other studies carefully and finally found a significant difference of sex and age between the case and the control in this study. The sex and age information for each study is listed in Table 4. Cases and controls matched by sex and age in most studies. However, in the study of Li et al. (2011), we observed a significant difference between the case and control both by sex (p<0.001) and age (p<0.001). Besides, sex ratios between the case and control group in the study of Hall et al. (2006) were not matched well (p<0.001). However, in the sensitivity analysis, this article did not influent the heterogeneity very much. This time, after omitting the Li et al. (2011), the overall heterogeneity between studies was not significant (P = 0.40, I^2^ = 3.8). These results proved that the study of Li et al. (2011) has a significant heterogeneity from other studies. After removing the study of Li et al. (2011), overall and subgroups ORs in 9 studies were calculated again in the recessive model. Results are summarized in Table S4. No significant heterogeneity was found in any subgroups. Results were in fixed models and the overall OR = 1.45 (95% CI 1.21–1.74, p<0.0001, Figure 4). Comparing the former subgroups analysis, results had some changes. As shown in Table S4, all results of subgroups reached significant levels. Then, a new sensitivity analysis was performed on the remained 9 studies. Before and after deleting each study, no significant heterogeneity between the remaining studies was found (Table S5). Forest plot of sensitivity analysis in the fixed model is shown in Figure S1. The outcomes were all similar after removing each study. These results suggest that no individual study significantly affected the overall OR in the new meta-analysis.

**Table 3:** Sensitivity analysis of 10 studies (recessive model).

	Fix Model		Random model		heterogeneity		
Study Omitted	OR(95%-CI)	p-Value	OR(95%-CI)	p-Value	tau^2^	I^2^(%)	p-Value
None	1.348(1.130,1.608)	0.001	1.366(1.062,1.756)	0.015	0.070	44.7	0.06
Hall 2006	1.318(1.099,1.580)	0.003	1.316(1.013,1.709)	0.040	0.070	45.7	0.06
Hao 2004	1.416(1.153,1.740)	0.001	1.404(1.047,1.884)	0.024	0.094	47.8	0.05
Hu 2010	1.257(1.040,1.520)	0.018	1.282(0.996,1.651)	0.054	0.053	36.8	0.12
Li 2011	1.451(1.207,1.744)	<0.0001	1.458(1.206,1.763)	<0.0001	0.003	3.8	0.40
Liu 2005	1.403(1.165,1.689)	<0.001	1.431(1.099,1.865)	0.008	0.069	44.1	0.07
Upadhyay 2010^a^	1.354(1.130,1.621)	0.001	1.377(1.050,1.805)	0.021	0.083	50.6	0.04
Upadhyay 2010^b^	1.322(1.103,1.586)	0.003	1.332(1.019,1.741)	0.036	0.078	48.6	0.05
Wang 2009	1.340(1.116,1.609)	0.002	1.357(1.029,1.790)	0.031	0.086	50.7	0.04
Xing 2001	1.303(1.080,1.572)	0.006	1.322(1.003,1.743)	0.048	0.080	47.5	0.05
Zhu 2009	1.333(1.105,1.607)	0.003	1.352(1.018,1.796)	0.037	0.090	50.5	0.04

a Kashmiri population;

b Uttar Pradesh population.

**Abbreviations:** OR: odds ratio; CI: confidence intervals; HWE: Hardy–Weinberg equilibrium.

**Table 4:** Sex and age information extracted from original articles.

First author	Year	Sex					Age		
		Case		Control		Result	Case	Control	Result
		Male	Female	Male	Female				
Hall	2006	713	98	831	252	p<0.001	<50∶ 140	<50∶ 203	p = 0.095
							50–54∶148	50–54∶170	
							55–59∶155	55–59∶178	
							60–64∶142	60–64∶174	
							65–69∶ 106	65–69∶ 162	
							70–74∶ 99	70–74∶ 149	
							≥75∶21	≥75∶47	
Hao	2004	N.A.	N.A.	N.A.	N.A.	match^a^	N.A.	N.A.	match^a^
Hu	2010	171	64	167	61	p = 0.907	61.60±10.06	61.57±10.17	p = 0.974
Li	2011	162	64	125	121	p<0.001	<50∶38	<50∶142	p<0.001
							50–60∶102	50–60∶66	
							≥60∶86	≥60∶38	
Liu	2005	52	54	52	54	p = 1.000	N.A.	N.A.	match^a^
Upadhyay	2010^b^	92	43	139	56	p = 0.541	60.38±8.40	57.98±12.66	p = 0.055
	2010^c^	147	53	152	55	p = 0.987	56.52±12.09	55.41±10.35	p = 0.300
Wang	2009	80	52	83	50	p = 0.763	N.A.	N.A.	match^a^
Xing	2001	140	56	143	58	p = 0.950	55.8±9.0	55.5±8.6	p = 0.734
Zhu	2009	105	83	126	77	p = 0.212	61.03	60.77	p = 0.061^a^

a Described in the original article;

b in Kashmiri population; c: in Uttar Pradesh population.

**Abbreviations:** N.A: Not Available.

### Cumulative Meta-analysis and Publication Bias

**Figure 5:**
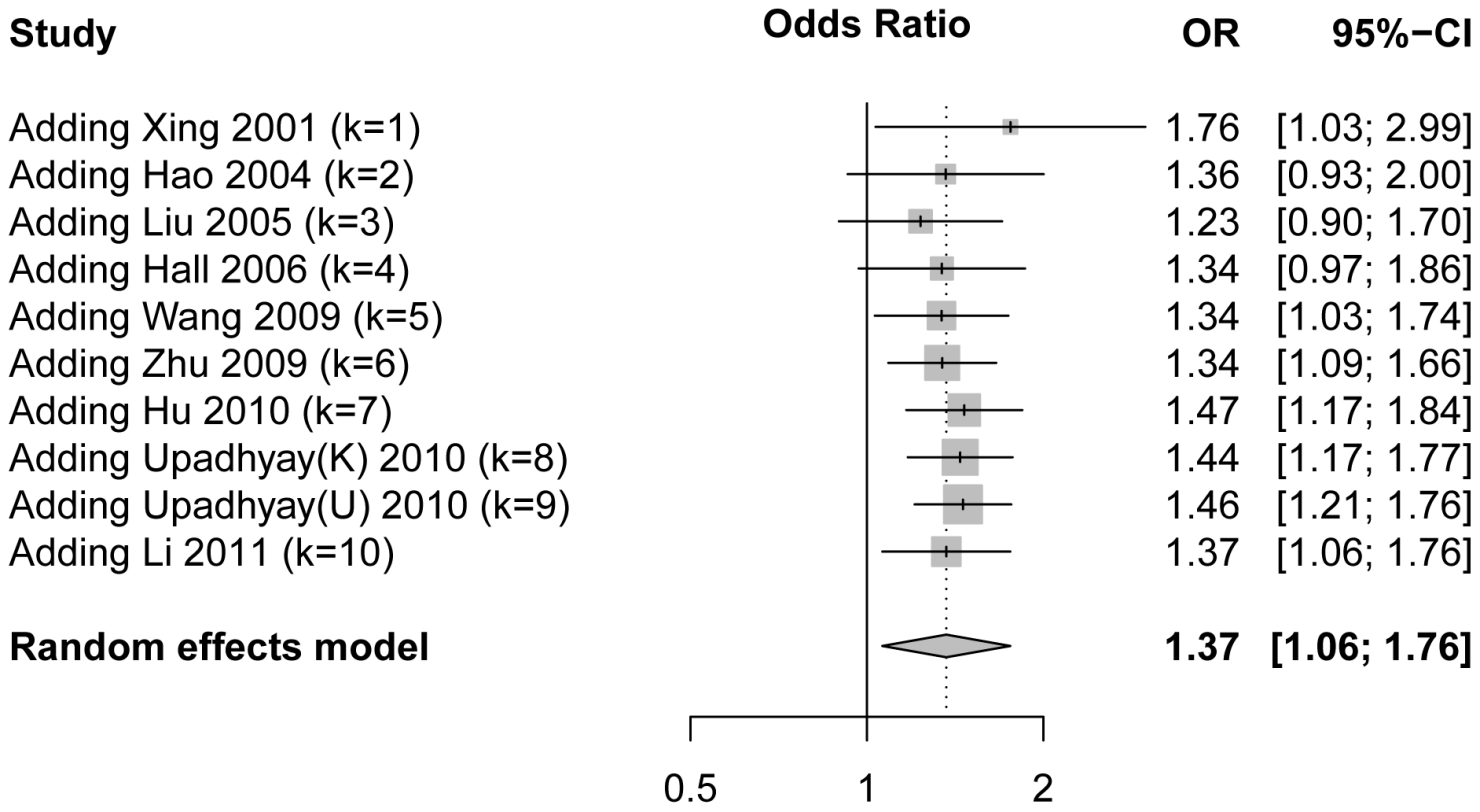
Forest plots of results of cumulative meta-analysis by published year in the recessive model. Pooled odds ratios (ORs) with 95% confidence limits (CIs) at the end of each information step were shown.

Cumulative meta-analysis of hOGG1 ser326cys with ESCC was conducted via the assortment of studies by publication time. Inclinations towards significant association were evident over time in the recessive model (Figure 5), but not in the dominant model (Figure S2). These results suggest that the precision of the estimates was progressively boosted by continually adding more samples.

**Figure 6:**
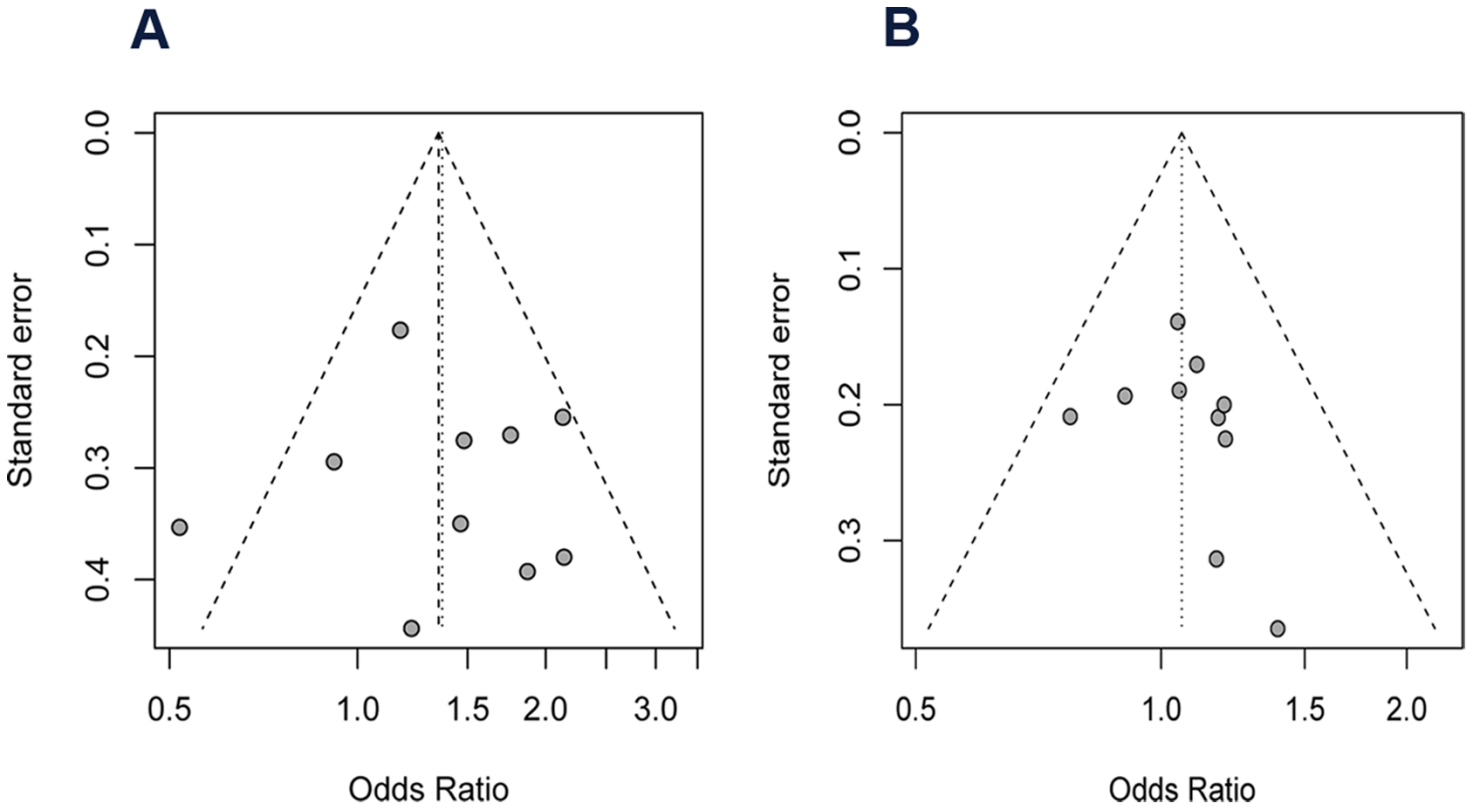
Begg’s funnel plot with 95% confidence limits (CIs) of publication bias test. (A). recessive model; (B). dominant model.

Funnel plot and Egger's test were performed to assess publication bias. The shapes of the funnel plots indicate that there was no obvious asymmetry. And the Egger's test also shows no publication bias (recessive: t = 0.27, p = 0.79; dominant: t = 0.91, p = 0.39; Figure 6).

## Discussion

Oxidative DNA damage occurs in a cell when the production of reactive oxygen species (ROS) exceeds the cell’s antioxidant-defense capacity, leading to cell apoptosis and producing mutations in the DNA [28], [29]. Among many factors of oxidative DNA damage, 8-hydroy-2-deoxyguanine (8-OHdG) is one of the most abundant oxidative products of highly mutagenic because of its propensities to mispair with adenine during DNA replication and to cause ultimately GC to TA mutation [30].

Many studies have reported that the hOGG1 gene could remove 8-OHdG from DNA by base excision repair (BER) pathway [31], [32]. If hOGG1 was dysfunctional, the damage could be left unrepaired, leading to mutations or carcinogenesis. A polymorphism of the hOGG1 has been identified, which caused an amino substitution from serine to cysteine in a codon 326. A number of studies have suggested that this variation may be associated with increased risks of several kinds of cancer. Several of them have focused on the association between the hOGG1 Ser326Cys polymorphism and esophageal cancer risk, but the results were inconclusive. Hence, this meta-analysis was needed to provide a quantitative approach for combining the different results.

The present meta-analysis includes 1987 esophageal cancer cases and 2926 controls. As far as we know, this is the first meta-analysis carried out with the aim of investigating the relationship between hOGG1 Ser326Cys polymorphism and ESCC risks. Results were significant in the recessive and additive model. Comparing with the Ser/Ser and Ser/Cys genotype, Cys/Cys genotype might contribute to increase the risk of ESCC. And the ESCC risk is positively correlated with the number of Cys allele.

When taking the sensitivity analyses, we found an article that inference the overall result very much and have heterogeneity to other studies. When we remove this study, the heterogeneity disappeared and the results became more stable. By comparing this article to others, we finally found the sex rate and age in the case group were significantly different from those in the control group. Some studies have found that ESCC most often occurs in men, and more often seen in aged 50–70 [33], [34]. These results suggest that when designing a case-control study of genetic polymorphism and cancer risk, investigator should match case and control well in sex rate and age, in order to give a reliable conclusion.

In many previous studies of meta-analysis, HWE-deviation studies were often ignored in the pooled estimate. However, a researcher argued that studies that appear to deviate from HWE should not be excluded unless there are other grounds for doubting the quality of the study [35]. In the sensitivity analysis of our research, no significant change of the OR was found when we remove the HWE-deviation study (Upadhyay 2010 “UP” Population). This result was consistent with that article. Therefore this study was included in our research.

In subgroup analysis by esophageal cancer types, we found that the hOGG1 Ser326Cys polymorphism was significantly associated with ESCC in the Caucasian population. However, the number of studies in this subgroup was a little small (n = 4), which may reduce the reliability of the results. Interestingly, we found a significant association between hOGG1 Ser326Cys polymorphism and ESCC in population controls group rather than controls based on the hospital. This finding suggests that Cys/Cys carrier may also cause other diseases risks in addition to ESCC.

In conclusion, our meta-analysis suggests that the hOGG1 Ser326Cys polymorphism is associated with esophageal cancer susceptibility. Cys/Cys carriers have more risk on ESCC rather than Ser/Ser and Ser/Cys carriers. Each copy of Cys modifies the risk of ESCC in an additive form. It is the homozygous Cys/Cys have a higher risk than heterozygous Cys/Ser. Moreover, in order to get a more reliable result, some factors such as gender and age should be matched in a case-control design study.

Several limitations of this meta-analysis should be addressed. First, we abandoned 2 studies when we read the full text of articles because they are mixed cancer types (EAC and ESCC); but we cannot get the numbers of ESCC patients from studies or authors. This limited the scale of the data to the meta-analysis. Second, genotypes errors may also influence the results, because the quality control of genotypic was not well documented in some studies. Third, several studies had a relatively small sample size.

In order to provide a more precise estimation, further research is necessary to use standardized unbiased homogenous cancer patients and well-matched controls to investigate the combined effects. That would lead to a better, comprehensive understanding of the association between hOGG1 Ser326Cys polymorphism and esophageal cancer risk.

## Supporting Information

Figure S1 **Sensitivity analysis without the study of Li et al.** Results were computed by omitting each study. Fixed-effects estimates were used. Each OR means the result when remove the corresponding study.
(PNG)

Figure S2 **Forest plots of cumulative analysis in the dominant model.** Pooled odds ratios (ORs) with 95% confidence limits (CIs) at the end of each information step were shown.
(PNG)

Table S1 **Overall and subgroup results in the dominant models.**
(DOC)

Table S2 **Pairwise comparison and P-valule in the additive models.**
(DOC)

Table S3 **Overall and subgroup analysis in the additive models.**
(DOC)

Table S4 **Results after removing the study of Li et al. in the recessive models.**
(DOC)

Table S5 **Sensitivity analysis of 9 studies in the recessive model.**
(DOC)

## References

[1] Umar SB, Fleischer DE (2008) Esophageal cancer: epidemiology, pathogenesis and prevention. Nat Clin Pract Gastroenterol Hepatol 5: 517–526. 18679388 10.1038/ncpgasthep1223

[2] He J, Gu D, Wu X, Reynolds K, Duan X, et al (2005) Major causes of death among men and women in China. N Engl J Med 353: 1124–1134. 16162883 10.1056/NEJMsa050467

[3] Mohrenweiser HW, Wilson DM, Jones IM (2003) Challenges and complexities in estimating both the functional impact and the disease risk associated with the extensive genetic variation in human DNA repair genes. Mutat Res 526: 93–125. 12714187 10.1016/s0027-5107(03)00049-6

[4] Dherin C, Radicella JP, Dizdaroglu M, Boiteux S (1999) Excision of oxidatively damaged DNA bases by the human alpha-hOgg1 protein and the polymorphic alpha-hOgg1(Ser326Cys) protein which is frequently found in human populations. Nucleic Acids Res 27: 4001–4007. 10497264 10.1093/nar/27.20.4001 PMC148667

[5] Audebert M, Radicella JP, Dizdaroglu M (2000) Effect of single mutations in the OGG1 gene found in human tumors on the substrate specificity of the Ogg1 protein. Nucleic Acids Res 28: 2672–2678. 10908322 10.1093/nar/28.14.2672 PMC102664

[6] Nishioka K, Ohtsubo T, Oda H, Fujiwara T, Kang D, et al (1999) Expression and differential intracellular localization of two major forms of human 8-oxoguanine DNA glycosylase encoded by alternatively spliced OGG1 mRNAs. Mol Biol Cell 10: 1637–1652. 10233168 10.1091/mbc.10.5.1637 PMC30487

[7] Kohno T, Shinmura K, Tosaka M, Tani M, Kim SR, et al (1998) Genetic polymorphisms and alternative splicing of the hOGG1 gene, that is involved in the repair of 8-hydroxyguanine in damaged DNA. Oncogene 16: 3219–3225. 9681819 10.1038/sj.onc.1201872

[8] Hall J, Hashibe M, Boffetta P, Gaborieau V, Moullan N, et al (2007) The association of sequence variants in DNA repair and cell cycle genes with cancers of the upper aerodigestive tract. Carcinogenesis 28: 665–671. 17040931 10.1093/carcin/bgl160

[9] Hao B, Wang H, Zhou K, Li Y, Chen X, et al (2004) Identification of genetic variants in base excision repair pathway and their associations with risk of esophageal squamous cell carcinoma. Cancer Res 64: 4378–4384. 15205355 10.1158/0008-5472.CAN-04-0372

[10] Li QD, Li H, Wang MS, Diao TY, Zhou ZY, et al (2011) Multi-susceptibility genes associated with the risk of the development stages of esophageal squamous cell cancer in Feicheng County. BMC Gastroenterol 11: 74. 21672255 10.1186/1471-230X-11-74 PMC3141752

[11] Upadhyay R, Malik MA, Zargar SA, Mittal B (2010) OGG1 Ser326Cys polymorphism and susceptibility to esophageal cancer in low and high at-risk populations of northern India. J Gastrointest Cancer 41: 110–115. 20069464 10.1007/s12029-009-9124-5

[12] Xing DY, Tan W, Song N, Lin DX (2001) Ser326Cys polymorphism in hOGG1 gene and risk of esophageal cancer in a Chinese population. Int J Cancer 95: 140–143. 11307145 10.1002/1097-0215(20010520)95:3<140::aid-ijc1024>3.0.co;2-2

[13] Handoll HH (2006) Systematic reviews on rehabilitation interventions. Arch Phys Med Rehabil 87: 875. 16731227 10.1016/j.apmr.2006.04.006

[14] Mantel N, Haenszel W (1959) Statistical aspects of the analysis of data from retrospective studies of disease. J Natl Cancer Inst 22: 719–748. 13655060

[15] DerSimonian R, Laird N (1986) Meta-analysis in clinical trials. Control Clin Trials 7: 177–188. 3802833 10.1016/0197-2456(86)90046-2

[16] Cochran WG (1954) SOME METHODS FOR STRENGTHENING THE COMMON X2 TESTS. Biometrics 10: 417–451.

[17] Armitage P (1955) TESTS FOR LINEAR TRENDS IN PROPORTIONS AND FREQUENCIES. Biometrics 11: 375–386.

[18] Greenland S, Longnecker MP (1992) Methods for trend estimation from summarized dose-response data, with applications to meta-analysis. Am J Epidemiol 135: 1301–1309. 1626547 10.1093/oxfordjournals.aje.a116237

[19] Egger M, Davey Smith G, Schneider M, Minder C (1997) Bias in meta-analysis detected by a simple, graphical test. BMJ 315: 629–634. 9310563 10.1136/bmj.315.7109.629 PMC2127453

[20] Ferguson HR, Wild CP, Anderson LA, Murphy SJ, Johnston BT, et al (2008) No association between hOGG1, XRCC1, and XPD polymorphisms and risk of reflux esophagitis, Barrett's esophagus, or esophageal adenocarcinoma: results from the factors influencing the Barrett's adenocarcinoma relationship case-control study. Cancer Epidemiol Biomarkers Prev 17: 736–739. 18349297 10.1158/1055-9965.EPI-07-2832

[21] Tse D, Zhai R, Zhou W, Heist RS, Asomaning K, et al (2008) Polymorphisms of the NER pathway genes, ERCC1 and XPD are associated with esophageal adenocarcinoma risk. Cancer Causes Control 19: 1077–1083. 18478337 10.1007/s10552-008-9171-4 PMC3106102

[22] Lagadu S, Lechevrel M, Sichel F, Breton J, Pottier D, et al (2010) 8-oxo-7,8-dihydro-2′-deoxyguanosine as a biomarker of oxidative damage in oesophageal cancer patients: lack of association with antioxidant vitamins and polymorphism of hOGG1 and GST. J Exp Clin Cancer Res 29: 157. 21134244 10.1186/1756-9966-29-157 PMC3004823

[23] Gao C, Sugimura H, Takezaki T, Wu J, Li Z, et al. (2001) hOGG1 Genotypes, Life Style and the Risk of Esophageal and Stomach Cancers. Bulletin of Chinese Cancer 10: 3. CNKI database. Available: http://en.cnki.com.cn/Article_en/CJFDTOTAL-ZHLU200109001.htm. Accessed 2013 May 8. [Article in Chinese].

[24] Hu H, Wu J, Jin Y, Zhang L, Gao G (2010) hOGG1 genotype with susceptibility to esophageal cancer in Northern Henan regions. Shandong Medical Journal 50: 2. Wanfang database. Available: http://d.wanfangdata.com.cn/Periodical_shandyy201032026.aspx. Accessed 2013 May 8. [Article in Chinese].

[25] Liu R, Yin L, Pu Y, Liu Y, Hu X, et al. (2005) Relationship between human 8-hydroxyguanine glycosylase Ser326Cys gene polymorphism and esophageal cancer. China Public Health 21: 3. CNKI database. Available: http://en.cnki.com.cn/Article_en/CJFDTOTAL-ZGGW200512011.htm. Accessed 2013 May 8. [Article in Chinese].

[26] Wang Y, Zhang C, Chen Y, Deng Y, Ma Y, et al. (2009) Relationship between hOGG1 gene polymorphism and the susceptibility of esophageal cancer in Kazakh nationality. Journal of Xinjiang Medical University 32: 4. CNKI database. Available: http://en.cnki.com.cn/Article_en/CJFDTOTAL-XJYY200905008.htm. Accessed 2013 May 8. [Article in Chinese].

[27] Zhu X, Zhang H, Du H, Hao Q, Wu X, et al. (2009) Association of hOGG1 Polymorphism Ser326Cys between the Susceptibility of Esophageal Cancer and Its Clinicopathological Characteristics. Journal of Environmental & Occupational Medicine 26: 4. CNKI database. Available: http://en.cnki.com.cn/Article_en/CJFDTOTAL-LDYX200902009.htm. Accessed 2013 May 8. [Article in Chinese].

[28] Roy D, Liehr JG (1999) Estrogen, DNA damage and mutations. Mutat Res 424: 107–115. 10064854 10.1016/s0027-5107(99)00012-3

[29] Yoshie Y, Ohshima H (1998) Synergistic induction of DNA strand breakage by catechol-estrogen and nitric oxide: implications for hormonal carcinogenesis. Free Radic Biol Med 24: 341–348. 9433910 10.1016/s0891-5849(97)00269-4

[30] Shibutani S, Takeshita M, Grollman AP (1991) Insertion of specific bases during DNA synthesis past the oxidation-damaged base 8-oxodG. Nature 349: 431–434. 1992344 10.1038/349431a0

[31] Boiteux S, Radicella JP (1999) Base excision repair of 8-hydroxyguanine protects DNA from endogenous oxidative stress. Biochimie 81: 59–67. 10214911 10.1016/s0300-9084(99)80039-x

[32] Boiteux S, Radicella JP (2000) The human OGG1 gene: structure, functions, and its implication in the process of carcinogenesis. Arch Biochem Biophys 377: 1–8. 10775435 10.1006/abbi.2000.1773

[33] Bahmanyar S, Zendehdel K, Nyren O, Ye W (2007) Risk of oesophageal cancer by histology among patients hospitalised for gastroduodenal ulcers. Gut 56: 464–468. 17005761 10.1136/gut.2006.109082 PMC1856842

[34] Johnell O, Kanis JA (2004) An estimate of the worldwide prevalence, mortality and disability associated with hip fracture. Osteoporos Int 15: 897–902. 15490120 10.1007/s00198-004-1627-0

[35] Minelli C, Thompson JR, Abrams KR, Thakkinstian A, Attia J (2008) How should we use information about HWE in the meta-analyses of genetic association studies? Int J Epidemiol 37: 136–146. 18037675 10.1093/ije/dym234

