# Supplementary figures and images for: A Meta-Analysis of the Association between the hOGG1 Ser326Cys Polymorphism and the Risk of Esophageal Squamous Cell Carcinoma
Source: PLoS One. 2013 Jun 6;8(6):e65742. doi: 10.1371/journal.pone.0065742 (PMC3675068; doi:10.1371/journal.pone.0065742)

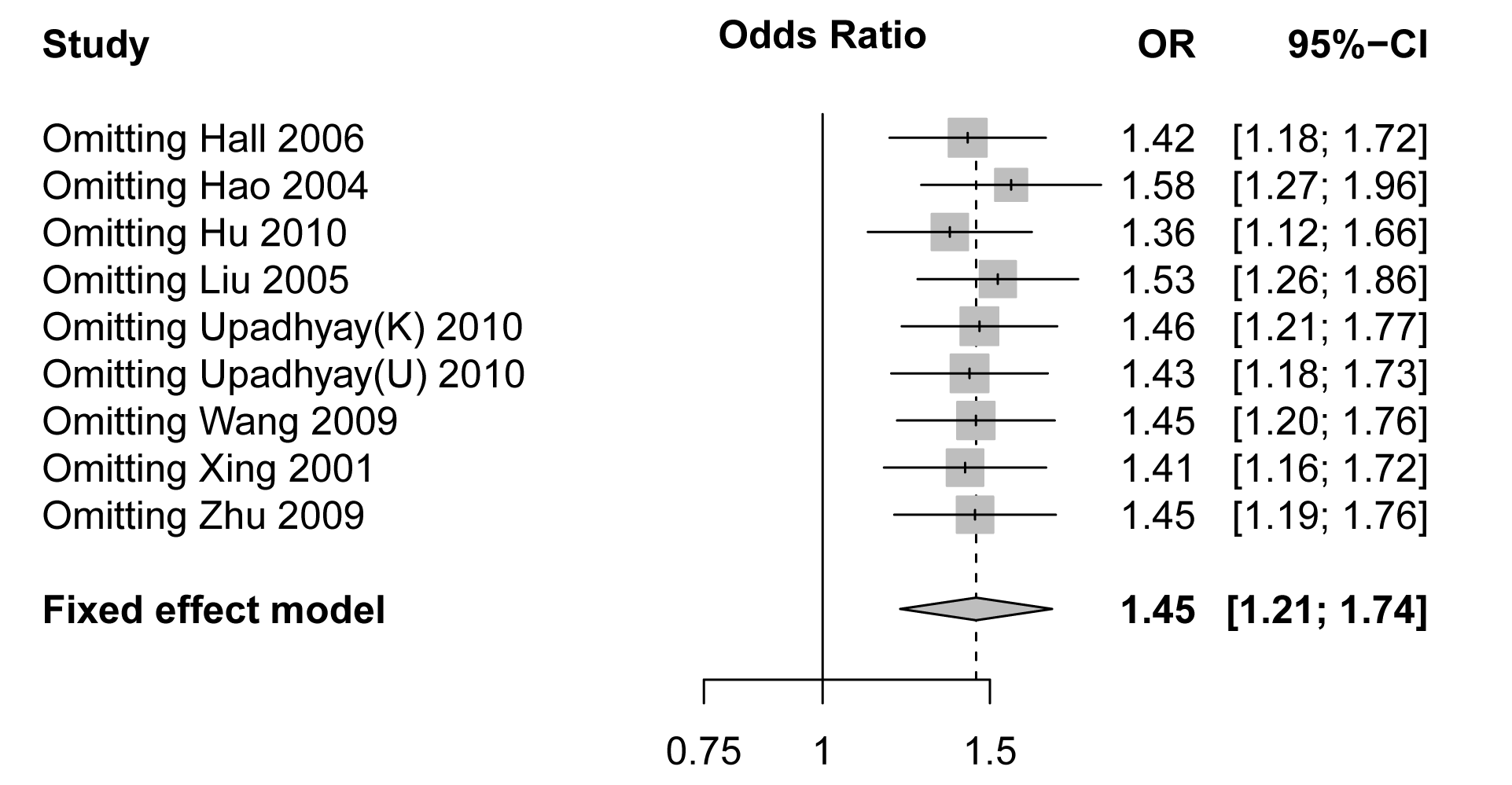

Supplement: Figure S1 — Sensitivity analysis without the study of Li et al. Results were computed by omitting each study. Fixed-effects estimates were used. Each OR means the result when remove the corresponding study. (PNG) [file pone.0065742.s001.png]

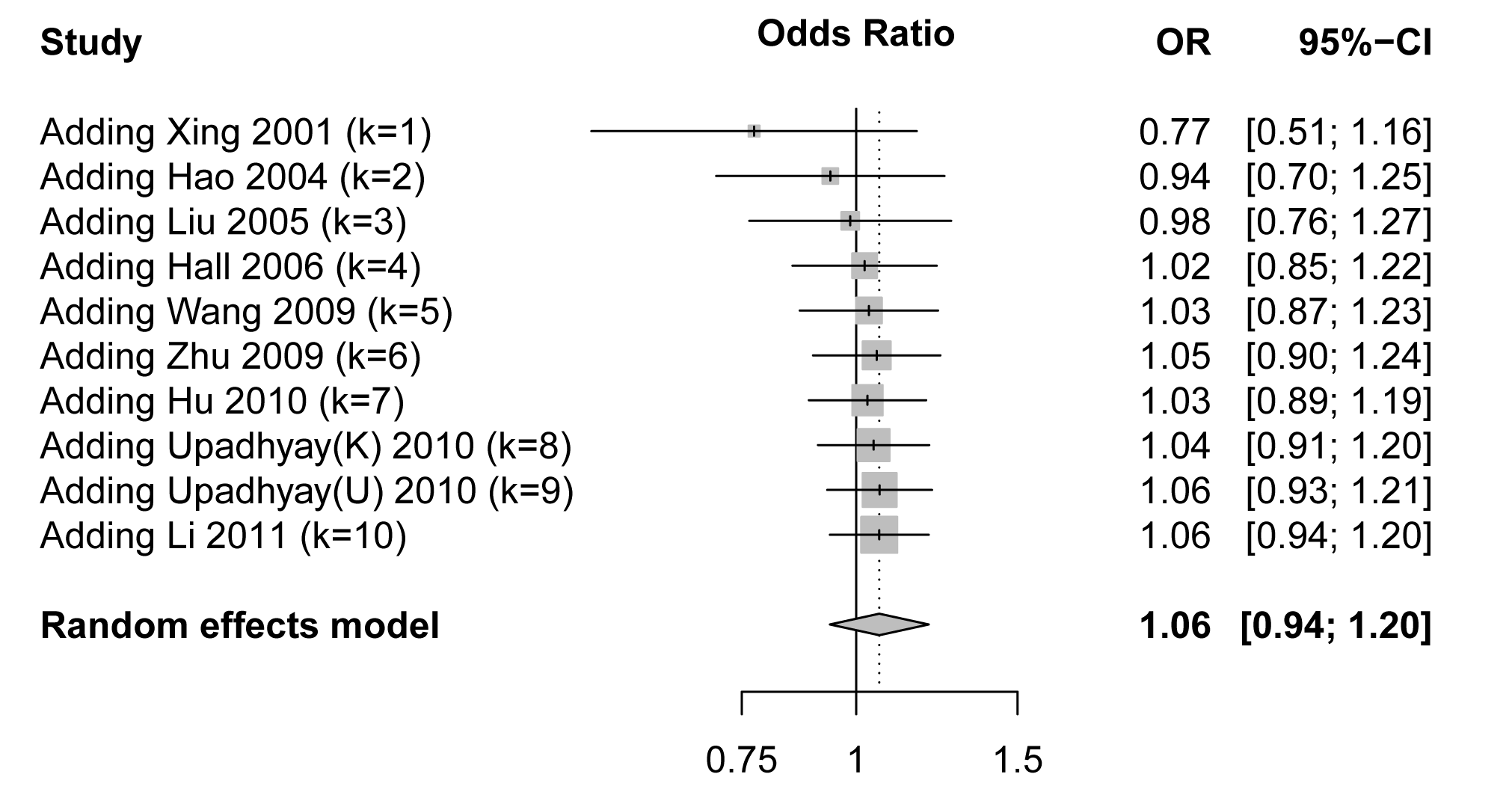

Supplement: Figure S2 — Forest plots of cumulative analysis in the dominant model. Pooled odds ratios (ORs) with 95% confidence limits (CIs) at the end of each information step were shown. (PNG) [file pone.0065742.s002.png]
