# Supplementary material for: A Meta-Analysis of the Association between the hOGG1 Ser326Cys Polymorphism and the Risk of Esophageal Squamous Cell Carcinoma
Source: PLoS One. 2013 Jun 6;8(6):e65742. doi: 10.1371/journal.pone.0065742 (PMC3675068; doi:10.1371/journal.pone.0065742)
Supplement: Table S1 — Overall and subgroup results in the dominant models. (DOC) [file pone.0065742.s003.doc]

**Quantitative analyses and the test of heterogeneity of the hOGG1 Ser326Cys polymorphism on the ESCC risk in** dominant models.

|  |  |  | **Q-test** | | |  |  |
| --- | --- | --- | --- | --- | --- | --- | --- |
|  | **Size^a^** | **Number** | **chi^2^** | ***p*** | **I^2^(%)** | **OR(95%CI)** | ***p*** |
| Overall | 4913 | 10 | 4.58 | 0.87 | 0 | 1.06(0.94,1.20) | 0.36 |
| Ethnic group |  |  |  |  |  |  |  |
| Asian | 2823 | 6 | 3.37 | 0.64 | 0 | 1.01(0.86,1.18) | 0.91 |
| Caucasian | 2090 | 4 | 0.12 | 0.99 | 0 | 1.16(0.94,1.42) | 0.17 |
| Published language |  |  |  |  |  |  |  |
| English language | 3636 | 6 | 3 | 0.7 | 0 | 1.05(0.91,1.22) | 0.49 |
| Chinese language | 1277 | 4 | 1.56 | 0.67 | 0 | 1.08(0.85,1.37) | 0.54 |
| Source of controls |  |  |  |  |  |  |  |
| Population | 2877 | 6 | 3.76 | 0.58 | 0 | 1.03(0.89,1.20) | 0.67 |
| Hospital | 2036 | 4 | 0.48 | 0.92 | 0 | 1.12(0.90,1.39) | 0.32 |
| Patient DNA source |  |  |  |  |  |  |  |
| Blood | 3662 | 7 | 0.91 | 0.99 | 0 | 1.12(0.96,1.29) | 0.14 |
| Tissue | 1251 | 3 | 2.05 | 0.36 | 2 | 0.93(0.74,1.18) | 0.56 |
| a: Sample size equals the total number of controls and cases. | | | | | | | |
| **Abbreviations:** OR, odds ratio; CI, confidence intervals. | | | | | | | |
