# Supplementary material for: A Meta-Analysis of the Association between the hOGG1 Ser326Cys Polymorphism and the Risk of Esophageal Squamous Cell Carcinoma
Source: PLoS One. 2013 Jun 6;8(6):e65742. doi: 10.1371/journal.pone.0065742 (PMC3675068; doi:10.1371/journal.pone.0065742)
Supplement: Table S2 — Pairwise comparison and P-valule in the additive models. (DOC) [file pone.0065742.s004.doc]

**Pairwise comparison and P-valule of Cochran-Armitage test in additive model**

| **Study** | **Compartion** | **OR(95%CI)** | ***P_trend_*** |
| --- | --- | --- | --- |
| Hall(2006) | Cys/Ser vs Ser/Ser | 1.02(0.72,1.44) | 0.24 |
|  | Cys/Cys vs Ser/Ser | 2.15(1.01,4.58) |  |
|  | Cys/Cys vs Cys/Ser | 2.12(0.97,4.61) |  |
| Hao(2004) | Cys/Ser vs Ser/Ser | 1.00(0.75,1.34) | 0.48 |
|  | Cys/Cys vs Ser/Ser | 1.17(0.80,1.71) |  |
|  | Cys/Cys vs Cys/Ser | 1.17(0.81,1.69) |  |
| Hu(2010) | Cys/Ser vs Ser/Ser | 0.70(0.47,1.06) | 0.21 |
|  | Cys/Cys vs Ser/Ser | 1.75(1.01,3.03) |  |
|  | Cys/Cys vs Cys/Ser | 2.49(1.46,4.23) |  |
| Li(2011) | Cys/Ser vs Ser/Ser | 1.16(0.79,1.69) | 0.53 |
|  | Cys/Cys vs Ser/Ser | 0.56(0.27,1.17) |  |
|  | Cys/Cys vs Cys/Ser | 0.49(0.24,0.99) |  |
| Liu(2005) | Cys/Ser vs Ser/Ser | 1.50(0.70,3.18) | 0.76 |
|  | Cys/Cys vs Ser/Ser | 1.24(0.55,2.77) |  |
|  | Cys/Cys vs Cys/Ser | 0.83(0.45,1.52) |  |
| Upadhyay(2010)^a^ | Cys/Ser vs Ser/Ser | 1.18(0.75,1.86) | 0.4 |
|  | Cys/Cys vs Ser/Ser | 1.33(0.54,3.27) |  |
|  | Cys/Cys vs Cys/Ser | 1.12(0.46,2.76) |  |
| Upadhyay(2010)^b^ | Cys/Ser vs Ser/Ser | 1.11(0.74,1.66) | 0.16 |
|  | Cys/Cys vs Ser/Ser | 1.97(0.89,4.39) |  |
|  | Cys/Cys vs Cys/Ser | 1.78(0.81,3.94) |  |
| Wang(2009) | Cys/Ser vs Ser/Ser | 1.06(0.55,2.01) | 0.33 |
|  | Cys/Cys vs Ser/Ser | 1.52(0.68,3.40) |  |
|  | Cys/Cys vs Cys/Ser | 1.43(0.70,2.94) |  |
| Xing(2001) | Cys/Ser vs Ser/Ser | 0.63(0.40,0.97) | 0.78 |
|  | Cys/Cys vs Ser/Ser | 1.36(0.76,2.43) |  |
|  | Cys/Cys vs Cys/Ser | 2.17(1.23,3.82) |  |
| Zhu(2009) | Cys/Ser vs Ser/Ser | 1.07(0.69,1.65) | 0.2 |
|  | Cys/Cys vs Ser/Ser | 1.54(0.85,2.77) |  |
|  | Cys/Cys vs Cys/Ser | 1.44(0.81,2.55) |  |
| Overall | Cys/Ser vs Ser/Ser | 0.99(0.87,1.12) | 0.02^c^ |
|  | Cys/Cys vs Ser/Ser | 1.34(1.10,1.63) |  |
|  | Cys/Cys vs Cys/Ser | 1.37(1.13,1.65) |  |
| a: Kashmiri population; b: Uttar Pradesh population; c: p-value of Greenland and Longnecker's method. | | | |
| **Abbreviations:** OR: odds ratio; CI: confidence intervals. | | | |
