# Supplementary material for: A Meta-Analysis of the Association between the hOGG1 Ser326Cys Polymorphism and the Risk of Esophageal Squamous Cell Carcinoma
Source: PLoS One. 2013 Jun 6;8(6):e65742. doi: 10.1371/journal.pone.0065742 (PMC3675068; doi:10.1371/journal.pone.0065742)
Supplement: Table S3 — Overall and subgroup analysis in the additive models. (DOC) [file pone.0065742.s005.doc]

**Overall and subgroup analysis in additive model**

|  | **Q-test** | | | |  |  |  |
| --- | --- | --- | --- | --- | --- | --- | --- |
|  | **chi^2^** | **df** | **p-Value** | **I^2^(%)** | **PCC** | **SE** | **p-Value** |
| Overall | 3.57 | 9 | 0.94 | 0 | 0.109 | 0.046 | 0.02 |
| Ethnic group |  |  |  |  |  |  |  |
| Asian | 2.14 | 5 | 0.83 | 0 | 0.074 | 0.055 | 0.18 |
| Caucasian | 0.08 | 3 | 1 | 0 | 0.19 | 0.084 | 0.02 |
| Language |  |  |  |  |  |  |  |
| English | 2.77 | 5 | 0.74 | 0 | 0.087 | 0.056 | 0.12 |
| Chinese | 0.31 | 3 | 0.96 | 0 | 0.156 | 0.081 | 0.055 |
| Source of control |  |  |  |  |  |  |  |
| Population | 1.38 | 5 | 0.93 | 0 | 0.12 | 0.055 | 0.03 |
| Hospital | 2.06 | 3 | 0.56 | 0 | 0.083 | 0.084 | 0.32 |
| DNA source |  |  |  |  |  |  |  |
| Blood | 2.89 | 6 | 0.82 | 0 | 1 | 0.056 | 0.07 |
| Tissue | 0.6 | 2 | 0.74 | 0 | 0.127 | 0.081 | 0.11 |
| **Abbreviations:** PCC: Pearson correlation coefficient; SE: standard error. | | | | | | | |
