# Supplementary material for: A Meta-Analysis of the Association between the hOGG1 Ser326Cys Polymorphism and the Risk of Esophageal Squamous Cell Carcinoma
Source: PLoS One. 2013 Jun 6;8(6):e65742. doi: 10.1371/journal.pone.0065742 (PMC3675068; doi:10.1371/journal.pone.0065742)
Supplement: Table S4 — Results after removing the study of Li et al. in the recessive models. (DOC) [file pone.0065742.s006.doc]

|  | **Q-test** | | | |  |  |  |
| --- | --- | --- | --- | --- | --- | --- | --- |
|  | **Chi^2^** | **df** | **p-Value** | **I^2^(%)** | **sample size^a^** | **OR(95%CI)** | **p-Value** |
| Overall | 8.32 | 8 | 0.4032 | 3.8 | 4442 | 1.45(1.21,1.74) | <0.0001 |
| Ethnic group |  |  |  |  |  |  |  |
| Asian | 6.57 | 4 | 0.1606 | 39.1 | 2352 | 1.40(1.14,1.73) | 0.002 |
| Caucasian | 1.16 | 3 | 0.763 | 0 | 2090 | 1.64(1.12,2.40) | 0.01 |
| Published language |  |  |  |  |  |  |  |
| English language | 3.59 | 4 | 0.464 | 0 | 3165 | 1.42(1.12,1.82) | 0.005 |
| Chinese language | 4.7 | 3 | 0.1953 | 36.1 | 1277 | 1.49(1.12,1.96) | 0.006 |
| Source of controls |  |  |  |  |  |  |  |
| Population | 4.75 | 5 | 0.4032 | 0 | 2877 | 1.50(1.22,1.85) | 0.0001 |
| Hospital | 3.25 | 2 | 0.1969 | 38 | 1565 | 1.29(0.88,1.89) | 0.2 |
| DNA source of cases |  |  |  |  |  |  |  |
| blood | 4.46 | 5 | 0.4848 | 4.46 | 3191 | 1.27(1.01,1.61) | 0.04 |
| tissue | 9.02 | 2 | 0.6217 | 9.02 | 1251 | 1.79(1.33,2.42) | 0.02 |
| a: Sample size equals the total number of controls and cases | | | | | | | |
| **Abbreviations:** OR, odds ratio; CI, confidence intervals | | | | | | | |

**Quantitative analyses and the test of heterogeneity of the hOGG1 Ser326Cys polymorphism on the ESCC risk after removing Li (2011) study in a recessive model.**
