# Supplementary material for: A Meta-Analysis of the Association between the hOGG1 Ser326Cys Polymorphism and the Risk of Esophageal Squamous Cell Carcinoma
Source: PLoS One. 2013 Jun 6;8(6):e65742. doi: 10.1371/journal.pone.0065742 (PMC3675068; doi:10.1371/journal.pone.0065742)
Supplement: Table S5 — Sensitivity analysis of 9 studies in the recessive model. (DOC) [file pone.0065742.s007.doc]

| **Sensitivity analysis of 9 studies(Recessive Model)** | | | | | |
| --- | --- | --- | --- | --- | --- |
|  | Fix Model | | Heterogeneity | | |
| Study Omitted | OR(95%-CI) | P-value | Tau^2^ | I^2^(%) | P-value |
| None | 1.451(1.207,1.744) | <0.0001 | 0.003 | 3.8 | 0.40 |
| Hall 2006 | 1.421(1.177,1.718) | 0.0003 | 0.002 | 2.9 | 0.41 |
| Hao 2004 | 1.580(1.270,1.963) | <0.0001 | 0.000 | 0.0 | 0.51 |
| Hu 2010 | 1.361(1.116,1.660) | 0.0023 | 0.000 | 0.0 | 0.58 |
| Liu 2005 | 1.530(1.259,1.858) | <0.0001 | 0.000 | 0.0 | 0.59 |
| Upadhyay 2010^a^ | 1.463(1.211,1.766) | <0.0001 | 0.013 | 14.2 | 0.32 |
| Upadhyay 2010^b^ | 1.428(1.181,1.726) | 0.0002 | 0.010 | 11.1 | 0.34 |
| Wang 2009 | 1.450(1.198,1.755) | 0.0001 | 0.015 | 15.8 | 0.31 |
| Xing 2001 | 1.412(1.161,1.719) | 0.0006 | 0.009 | 9.7 | 0.35 |
| Zhu 2009 | 1.447(1.190,1.760) | 0.0002 | 0.016 | 15.8 | 0.31 |

a: Kashmiri population; b: Uttar Pradesh population
